# 'PACLIMS': A component LIM system for high-throughput functional genomic analysis

**DOI:** 10.1186/1471-2105-6-94

**Published:** 2005-04-12

**Authors:** Nicole Donofrio, Ravi Rajagopalon, Douglas Brown, Stephen Diener, Donald Windham, Shelly Nolin, Anna Floyd, Thomas Mitchell, Natalia Galadima, Sara Tucker, Marc J Orbach, Gayatri Patel, Mark Farman, Vishal Pampanwar, Cari Soderlund, Yong-Hwan Lee, Ralph A Dean

**Affiliations:** 1 Department of Plant Pathology, Fungal Genomics Laboratory, North Carolina State University, Raleigh, NC, USA; 2 Department of Plant Pathology, University of Arizona, Tucson, AZ, USA; 3 Department of Plant Pathology, Plant Sciences Building, 1405 Veteran's Drive, University of Kentucky, Lexington, KY, 40546, USA; 4 Arizona Genomics Computational Laboratory, University of Arizona, Tucson, AZ, USA; 5 School of Agricultural Biotechnology, Seoul National University, Seoul, Korea

## Abstract

**Background:**

Recent advances in sequencing techniques leading to cost reduction have resulted in the generation of a growing number of sequenced eukaryotic genomes. Computational tools greatly assist in defining open reading frames and assigning tentative annotations. However, gene functions cannot be asserted without biological support through, among other things, mutational analysis. In taking a genome-wide approach to functionally annotate an entire organism, in this application the ~11,000 predicted genes in the rice blast fungus (*Magnaporthe grisea*), an effective platform for tracking and storing both the biological materials created and the data produced across several participating institutions was required.

**Results:**

The platform designed, named PACLIMS, was built to support our high throughput pipeline for generating 50,000 random insertion mutants of *Magnaporthe grisea*. To be a useful tool for materials and data tracking and storage, PACLIMS was designed to be simple to use, modifiable to accommodate refinement of research protocols, and cost-efficient. Data entry into PACLIMS was simplified through the use of barcodes and scanners, thus reducing the potential human error, time constraints, and labor. This platform was designed in concert with our experimental protocol so that it leads the researchers through each step of the process from mutant generation through phenotypic assays, thus ensuring that every mutant produced is handled in an identical manner and all necessary data is captured.

**Conclusion:**

Many sequenced eukaryotes have reached the point where computational analyses are no longer sufficient and require biological support for their predicted genes. Consequently, there is an increasing need for platforms that support high throughput genome-wide mutational analyses. While PACLIMS was designed specifically for this project, the source and ideas present in its implementation can be used as a model for other high throughput mutational endeavors.

## Background

Genome sequencing is the first step towards understanding the complex interplay between pathways and networks that determine the biology of living organisms. The next important step in these analyses is to perform genome-wide investigations to identify the functions of individual genes. While hybridization techniques such as DNA-based microarrays can provide insight into groups of genes that potentially operate in common pathways, validation is required before final functional assignment [1]. Furthermore, many genes are regulated in a post-transcriptional manner, thus their function would not be definable by microarrays [2]. Genome-wide screens of mutants created by targeted and random mutagenesis, as well as the method of gene silencing, are particularly powerful for ascribing phenotypes to individual genes and gene families and can potentially validate predictions from sequence and microarray data [3-7].

In many cases, taking a genome-wide approach to functional gene analysis requires the combined skills and resources of several research groups working with a semi-automated, rapid-throughput pipeline. To facilitate our goal of a comprehensive functional gene analysis in the fungus *Magnaporthe grisea*, we have developed a platform for high-throughput mutagenesis and phenotypic characterization. Using this platform, we are seeking to elucidate the functions of the approximately 11,000 genes in the thirty-eight megabase genome of this fungus [8]. *M. grisea *is the causal agent of rice blast disease, the most devastating disease of rice worldwide [9]. The economic importance of this pathogen and its genetic tractability make it a model system for understanding fungal biology, as well as plant-pathogen interactions [10].

One of the strategies that we have adopted to determine the functions of individual genes is to create 50,000 *M. grisea *strains, each carrying a single random mutation within the genome. The mutant strains are generated by introducing a disruption cassette into the fungus, which consists of a DNA fragment that confers resistance to the antibiotic, hygromycin B [11]. Transformed *M. grisea *cells that incorporate the cassette into their chromosomal DNA are then able to grow on media containing the antibiotic. During the process, the cassette will often insert into an open reading frame or regulatory region, resulting in a loss of gene function and thus a biochemical or structural deficiency. Identification and characterization of phenotypic changes in each mutant provides information about the normal biological role(s) of the disrupted gene, whose identity is established by taking advantage of the fact that it has been "tagged" by the inserted antibiotic resistance marker [12,13].

Research groups from two universities, University of Arizona (UA) and University of Kentucky (UKY), are cooperating to create the tagged *M. grisea *lines and to characterize any phenotypic changes. The mutant strains are then shipped to North Carolina State University (NCSU), where they are screened for changes in pathogenicity using susceptible rice varieties. Finally, all mutant strains are sent to the Fungal Genetics Stock Center (Kansas City, MO), a fungal strain repository, where they will be archived and made available to the public. The distribution of research efforts and pooling of the resources and data generated dramatically increases the necessity of having a system for each research laboratory to enter and access the information being produced.

From creation to final analysis, each mutant is processed through a total of eight barcoded steps and four phenotypic assays resulting in the capture of a dozen individual pieces of data over a period of 3–6 months. The ability to log, process and archive information in an efficient and secure manner is vital to the success of this project. To record data and track these mutants, we have developed a minimal Laboratory Information Management System (LIMS), called PACLIMS (Phenotype Assay Component LIMS) that is described in this report. This system was designed to be flexible in order to accommodate the experimental protocol as it evolved. The software fulfills the role of process control by enforcing the steps of our protocol, and reduces laboratory and data entry errors while allowing the data generated at the three universities to be entered from separate locations.

**Figure 1:**
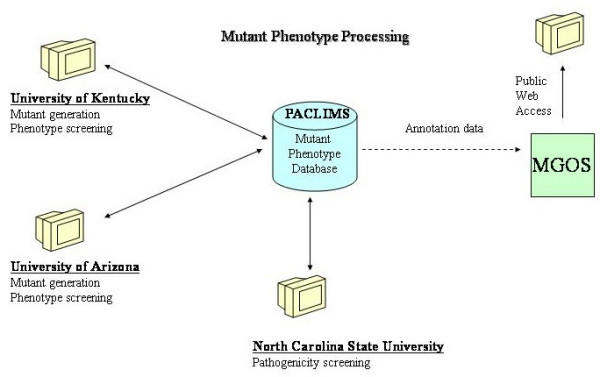
Schematic of data acquisition and transfer to PACLIMS from multiple universities.

Many LIMS are implemented using expensive commercial products or are integrated systems that provide a complete solution and utilization of commercial database systems [14-16]. A primary goal in the creation of PACLIMS was to design a system that simplified data entry, and was inexpensive yet flexible to allow modification based on user experience. PACLIMS utilizes the freely-available, standards-based SQL, HTML and SSL technologies, and adheres to common web practicesthroughout. Data is entered into PACLIMS by researchers working at each site (Figure 1), and the results from assays performed at each university are made available and updated on a daily basis through a publicly-accessible database called MGOS (*M. grisea*-*Oryza sativa*)[17]. In this paper, we describe the conception, creation and implementation of the PACLIMS database, as well as the experimental procedure and data it was designed to manage. Within the project website we provide access to a publicly available 'demo' database, documentation and the PACLIMS software which can be downloaded and modified to suit other researchers' needs.

## Implementation

The PACLIMS system was implemented with Open Source, freely available software. The server machine runs Red Hat linux (RH), which runs on a large variety of commodity PC hardware. The RH distribution includes most of the software components that are required to construct PACLIMS. The Postgresql relational database system was used for data storage [18]. This allows the utilization of transactions for data integrity, network based access, and supports numerous interface technologies. The Apache web server was employed for the user interface and for interconnecting the database and control programs, via a simple CGI oriented mechanism that follows normal web practices [19]. Implementation was performed using Perl, a common bioinformatics language, allowing the system to be readily modified [20,21].

### Distributed operations and client/server web interface

A centralized, web-based client/server paradigm was chosen to reduce the management burden presented by the system. All server-based processing occurs on a single computer. Web server dependence was minimized by using a simple CGI interface between the server and the PACLIMS control programs. Secure access is ensured by employing the SSL-based HTTPS protocol. Secure user-access and presentation of security credentials occurs through a web browser such as Netscape and Internet Explorer, so that when a user logs into the system, their identity is associated with all subsequent actions.

## Results

**Figure 2:**
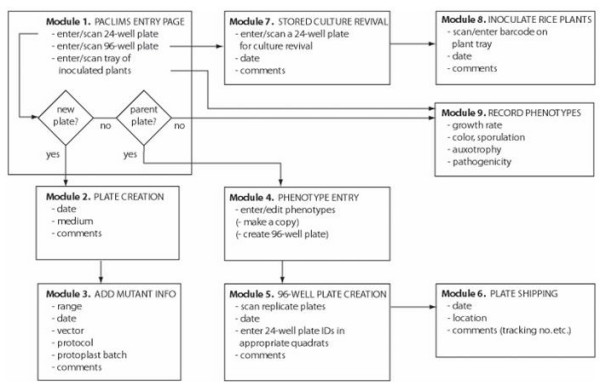
A schematic overview of the flow in information and materials managed by PACLIMS. **Module 1: **entry point for PACLIMS, accessed by scanning or entering a plate or plant rack's barcode; the user is directed to the appropriate web form. **Modules 2 **and **3: **displays web forms for data entry on a new, or "parent" plate, including fields for information on mutants in each of the 24 wells. **Module 4: **provides access to a web form for entry of growth rate data that is collected from the parent plate and allows the user to create "copies" of the parent plate for assaying other phenotypes. **Module 5: **guides and documents the transfer of paper disks from four 24-well "parent" plates into the four quadrants of three 96-well plates, used for permanent storage and shipping, the latter process being recorded with **Module 6**. **Module 7: **records the revival of cultures from permanent storage, specifically the reversion of the 96-well format into four 24-well plates for pathogenicity assays. **Module 8: **directs the inoculation process for each 24-well plate of spores, beginning with entering a barcode for a rack of plants to be inoculated, and culminating in entering pathological data for each mutant isolate. **Module 9: **records phenotypic data via four different web forms, each of which records specific phenotypes; module 1 controls the particular data entry form that is accessed.

PACLIMS is composed of nine modules that facilitate the management of three basic components of this project: barcoding for tracking the progress of mutants through the pipeline, mutant production and initial characterization, and pathogenicity screening (Figure 2). The role of PACLIMS in managing these processes is described below.

### Barcode management

**Figure 3:**
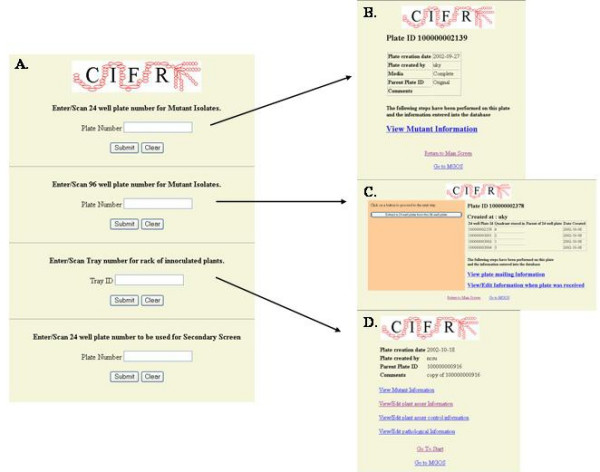
(A) Front page for barcode scanning into PACLIMS. (B-D) Depending upon which barcode has been scanned, the user will be transferred by Module 1 to the next step in the experimental process.

Due to the high-throughput nature of this project, all stages of mutant processing and analysis are performed in either 24- or 96-well microtiter plate format, with each plate being assigned a barcode. Thus, each mutant is identified by its barcode-assigned plate number and by its coordinates within the plate. The researcher uses a PACLIMS web-link to request sheets of barcode labels, which can be printed locally. To ensure that each plate has a unique identifier, PACLIMS controls the generation of barcode images, so that each barcode is printed only once. The researcher affixes a barcode to each microtiter plate and then scans it into PACLIMS (Figure 3A), whereupon the barcode identifier is permanently associated with that plate (Figure 3B and 3C). By separating barcode label generation and the association of a barcode with a plate, issues such as lost, misapplied and damaged labels, are avoided. If a previously used label is erroneously affixed to a new plate, the system recognizes that the barcode has already been assigned to a previous plate, and instructs the researcher to choose another barcode and re-enter the plate identifier. All copies of the parent plate and the derived (replicate) plates also receive barcodes and are scanned into the database. The barcode of any replicate plate can be re-scanned at any stage, including mutant production or pathogenicity screening, to trace its history back to the corresponding parent plate.

### Mutant production pipeline

The initial stages of mutant production and morphological characterization are performed at UKY and UA. After the creation of mutants and genetic purification each mutant is transferred to a well of a 24-well plate containing complete medium agar plus hygromycin with three cellulose paper disks on the agar surface. This "parent plate" marks the entry point for PACLIMS. All subsequent daughter plates can be tracked back to their parent. A barcode is attached to the plate and scanned into PACLIMS, which then directs the user through web forms, in order to record details about the plate's contents (Figure 3A and 3B). The parent plate is incubated for a defined period of time at which point the user collects phenotype data such as growth rate and enters it into the system (Figure 2, Module 9). PACLIMS also directs the user to create other copies of the mutants for sporulation and auxotrophy analyses. Permanent stocks are created in triplicate (Figure 2, Module 5), with one replicate being retained at the site of origin, one being shipped to NCSU for pathogenicity screening, and the final replicate going to the Fungal Genetics Stock Center (Kansas City, MO) for public request (Figure 2, Module 6). PACLIMS is used to direct the creation of these stocks, and to record the receipt of their shipment. All phenotype data generated are recorded in PACLIMS (Figure 2, Module 4, Module 9).

### Pathogenicity screening

**Figure 4:**
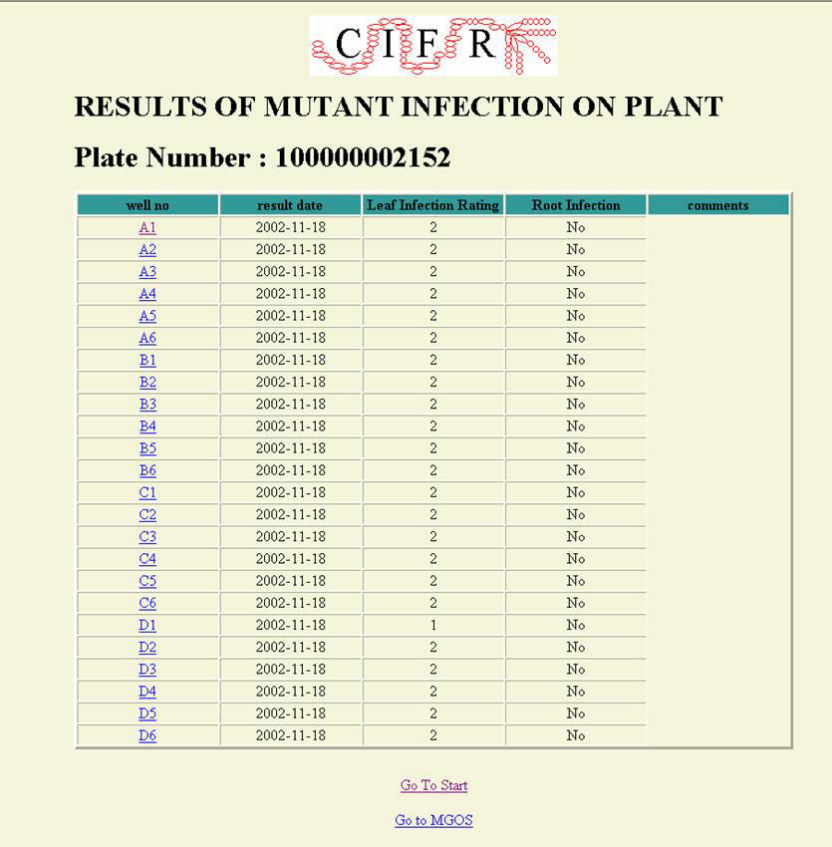
Data entry page for pathogenicity screen results.

Upon receipt of mutant plates by NCSU the barcode on the 96-well plate is scanned and PACLIMS logs the plates' arrival (Figure 3A) and provides a screen to "create" 24-well plates for "activation" of the cultures in the 96-well storage plate (Figure 2; Module 7). These 24-well plates are then used to generate conidia for pathogenicity assays and mycelia for DNA extractions. Each of these plates receives a barcode, and when they are scanned into the database, the user is automatically transferred to the corresponding stage of the experimental procedure. Mutants are screened for pathogenicity and each result is recorded in the PACLIMS database. Data entry is facilitated by scanning the barcode for the rack of inoculated plants, at which stage the user is presented a display of data columns set to the default value of wild-type for the individual wells (Figure 4). Mutants with aberrant phenotypes are re-tested in a secondary assay to reduce isolation of false positives after being transferred to a new 24-well plate consisting of only reduced pathogenicity mutants by the LIMS.

### Report generation

Sufficient reporting functionality is built into the system to support the data entry process. Contextual information is supplied to the user to allow review of the entered information prior to permanently committing it to the database. Robust reporting is provided by third party software such as Microsoft Access database communications protocol or database systems like MGOS by using Postgresql's own network communications protocol [17,18]. Separating and relegating reporting to an external component increases the reusability and component nature of the implementation. PACLIMS can be readily modified to account for different research protocols without disrupting the reporting mechanism. Moreover, specialized third party reporting tools provide a ready means of creating custom reports, as need dictates.

## Availability

The current version of PACLIMS is freely available to academic and non-profit users at . Furthermore, the system is modular and readily customized to suit a laboratory's specific needs for a high-throughput screen. There is no need for purchasing additional software to use the system. Laboratory personnel who have introductory level experience with Perl can readily adapt the software to different protocols. Please contact Ralph_Dean@ncsu.edu for further details.

## Authors' contributions

RR, DB, DW and VP coded the software, ND and SD wrote and edited the manuscript, SN, AF, NG, ST, and GP provided testing and feedback, RD, YL, CS, MF, MO, and TM developed the concept and provided guidance.
